# Polymyositis: A Rare Cause of Acute Respiratory Failure and a Diagnostic Dilemma

**DOI:** 10.7759/cureus.43887

**Published:** 2023-08-21

**Authors:** Kunjal Luhadia, Thanmay Sathi, Kanica Yashi, Megha Dogra

**Affiliations:** 1 Internal Medicine, Bassett Healthcare, Cooperstown, USA; 2 Internal Medicine, Saint Vincent Hospital, Worcester, USA

**Keywords:** diaphragm dysfunction, diseases of muscular system, autoimmune diseases, idiopathic polymyositis, polymyositis

## Abstract

Polymyositis is an autoimmune multisystemic disorder that affects the body's muscular system. It usually affects the proximal muscles of the shoulder, pelvis, neck flexor muscles, and sometimes, the hip extensor muscles. However, it can also affect the diaphragm causing acute respiratory failure. This case report educates clinicians about the atypical presentation of polymyositis, diagnosis, and treatment of this disease. It also emphasizes the importance of looking for an alternative diagnosis such as polymyositis when the treatment for the more common diagnoses such as community-acquired pneumonia does not improve the patient's respiratory status.

## Introduction

Polymyositis is an autoimmune multisystemic disorder that affects the body's muscular system. It usually affects the proximal muscles of the shoulder, pelvis, neck flexor muscles, and sometimes, the hip extensor muscles [1]. This condition develops due to the abnormal activation of cytotoxic T lymphocytes and macrophages against muscular antigens, damaging the skeletal muscles' endomysium [1-3]. Indirect damage can happen due to cytokines and interleukins [1].

The presenting features of polymyositis include progressive symmetric disease involving proximal girdle muscles and neck flexors that can sometimes be painful [1,4]. Constitutional symptoms include low-grade fever, anorexia, arthralgia, and weight loss. For the diagnosis, a complete physical exam with a detailed neuromuscular exam is critical to appreciate the muscles involved and the severity of the muscle weakness. Muscle enzymes such as creatine phosphokinase (CPK), lactate dehydrogenase (LDH), and aspartate aminotransferase (AST)/alanine transaminase (ALT) will be elevated. Further rheumatologic workup that may support the diagnosis if positive include anti-Jo-1, anti-Ro/Sjögren's syndrome (SS)A, anti-La/SSB, anti-Smith (Sm), anti-ribonucleoprotein (RNP) antibodies, anti-polymyositis (PM)-Scl, and anti-Ku antibodies, antinuclear antibody (ANA) [5]. Further workup would include an electromyography (EMG) and muscle MRI; a muscle biopsy would be required for a definite diagnosis. Some differential diagnoses include but are not limited to antisynthetase syndrome, inclusion body myositis, overlap syndrome, drug-induced myopathy, etc. Once the diagnosis is made, patients must get evaluated for interstitial lung disease and screened for malignancy, cardiac involvement, and esophageal dysfunction. The initial therapy includes glucocorticoids and immunomodulators such as azathioprine or methotrexate [5].

## Case presentation

A 26-year-old female patient was brought to the emergency department from a residential care facility for adults with developmental disabilities. The patient had a past medical history of seizure disorder, Bart Pumphrey syndrome, Klippel-Feil syndrome, and hypertension, and she was non-verbal at baseline. The patient was accompanied by an aide who said that the patient had been having shortness of breath associated with tachycardia and reduced oxygen saturation in the 80s for five months. It gradually progressed to the patient having breathlessness at rest and rapid breathing. Chest x-ray revealed patchy and streaky densities, more significant on the right side than on the left. Additionally, laboratory findings also showed elevated hepatic enzymes.

**Figure 1:**
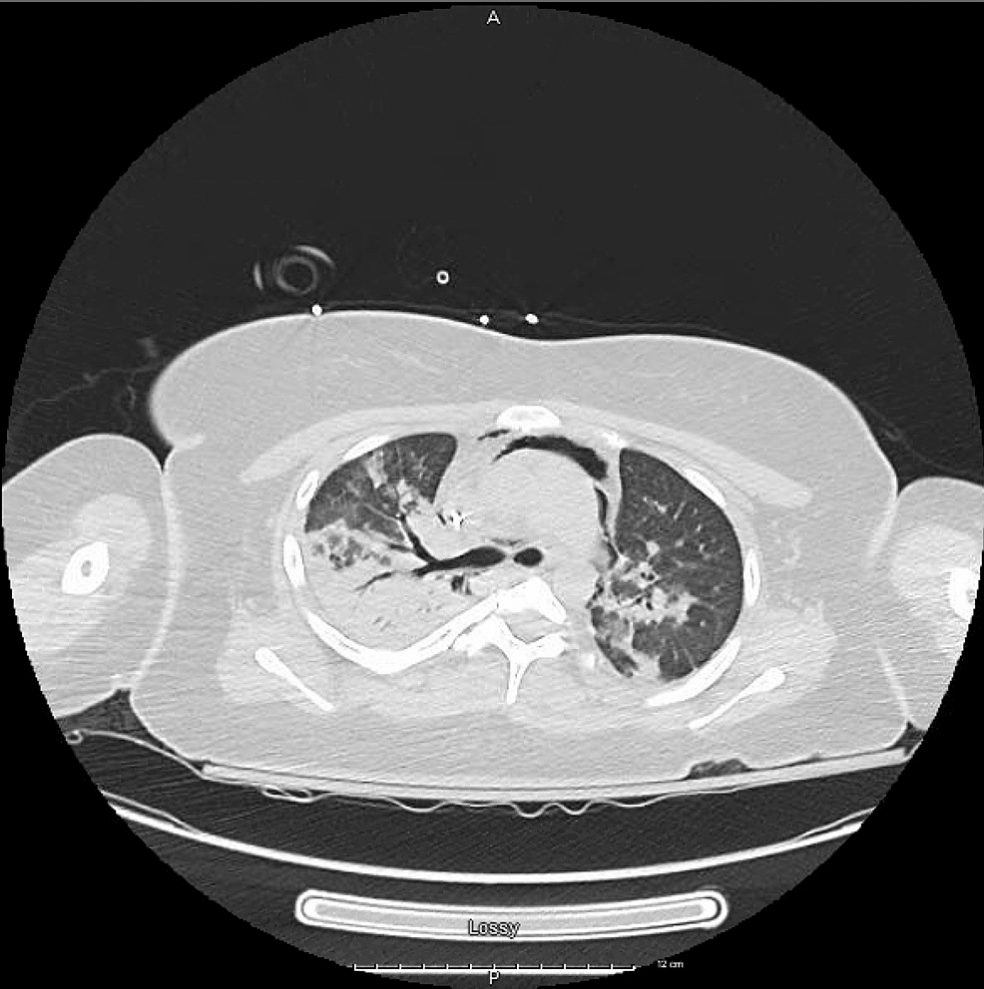
CT scan of the lungs showing bibasilar consolidation

The patient's acute symptoms were thought to be due to community-acquired pneumonia, and empiric antibiotic therapy was started with ceftriaxone and azithromycin after collecting blood culture and sputum culture. CT angiography of the chest imaging ruled out pulmonary embolism. However, it revealed bibasilar consolidation with subsegmental atelectasis (Figure 1); findings were also present on the previous CT scan obtained three months back. CT also showed a dilated esophagus, which was thought to be a risk factor for aspiration. The patient's vital signs data during her admission to the hospital are given in Table 1.

**Table 1:** Vital signs

Temperature	Afebrile
Blood pressure	94/49-130/86 mmHg
Respiratory rate	22-31 breaths/minute
Heart rate	58-110 beats/minute

After two days of receiving care on the hospital floor, the patient developed acute respiratory distress associated with hypotension, confusion, and agitation. Stat arterial blood gas was acquired, which showed a pH of 7.19 with hypercapnia and hypoxia. The patient was intubated and placed on mechanical ventilation. While staff were preparing for the procedure, the patient developed sudden bradycardia along with further worsening of hypoxia, which warranted initiation of cardiopulmonary resuscitation. Cardiopulmonary resuscitation (CPR) was performed, which resulted in a return of spontaneous circulation. Norepinephrine infusion was started via a central line to support blood pressure. Intravenous steroids were administered as a systemic inflammatory response was suspected. The patient was transferred to the intensive care unit (ICU).

Diagnostic bronchoscopy in the ICU revealed bloody secretions in both mainstem bronchi; the left upper lobe had erythematous bleeding mucosa. Samples were collected and sent for cytology and culture, which did not reveal any abnormal findings or infectious etiology. The patient’s symptoms were thought to be due to a combination of cryptogenic organizing pneumonia, obstructive sleep apnea, and aspiration pneumonitis. She was placed on pressure-regulated volume control (PRVC) ventilation. Steroids were continued due to suspicion of cryptogenic organizing pneumonia. GI was consulted for the dilated esophagus, which could be causing recurrent aspiration. An upper GI endoscopy was performed for suspicion of achalasia, but it was found to be normal. The patient failed two extubation attempts and had to be re-intubated. She was later slowly weaned off the ventilator with a tracheostomy tube placed and successive T-piece trials. A percutaneous gastrostomy tube was inserted for nutritional supplementation.

The patient’s breathing gradually improved and stabilized. She was downgraded from the ICU to the general medicine floor team with a trach collar for supplemental oxygen. At this time, her hepatic function panel, which was persistently elevated during hospitalization, was worked up. Laboratory tests for diagnosing autoimmune conditions were sent, along with other tests. The CPK and aldolase were found to be elevated, along with a high positive ANA nucleolar pattern with a positive PM Scl-70 antibody. Rheumatology was consulted and recommended starting the patient on prednisone 60 mg daily with subsequent laboratory tests (CPK, aldolase, erythrocyte sedimentation rate (ESR), C-reactive protein (CRP), and complete blood count (CBC)) in a few weeks to follow up on disease progress. Diaphragmatic weakness resulting from polymyositis contributing to the patient’s respiratory failure was considered at this point. It was also thought dysphagia was occurring due to muscle weakness leading to recurrent aspirations. The patient was eventually discharged to a skilled nursing facility for aggressive physical therapy due to her prolonged hospital stay.

She followed up with outpatient rheumatology for continuing care, and prednisone 60 mg was continued. After about a month, the patient was discharged from the rehabilitation facility and moved out of the area. She returned for a follow-up visit after about two years. During the visit, the patient appeared to be doing well and was quite active during the encounter. She displayed no signs of muscle weakness, and her swallowing returned to normal. On the musculoskeletal exam, the patient appeared to have full strength. During the clinic visit, she reported taking prednisone 25 mg since she moved out of the area and was still on that dose. Her polymyositis was no longer active. A plan was made to taper down her prednisone gradually to 10 mg. Currently, she is found to be doing well and is regularly following rheumatology for continued treatment.

## Discussion

Polymyositis usually affects people above the age of 20 years. This condition is more prevalent in Blacks than in Whites [6]. Different respiratory complications arise from polymyositis; interstitial lung disease is the most common [7-9]. Other complications include aspiration pneumonia, ventilatory insufficiency, and alveolar hypoventilation due to muscle weakness [7].

A study showed that in patients 45 years and younger, the risk of acute respiratory failure was found to be 2.11 times greater than in patients aged 45-64 years and 9.11 times greater in those who were 65 years or older, showing that younger patients are more likely to develop this complication. The study also showed that polymyositis patients with comorbidities like hypertension, chronic obstructive pulmonary disease (COPD), and peripheral vascular disease are at higher risk of developing acute respiratory failure [10,11].

This case report emphasizes the importance of looking for an alternative diagnosis when the treatment for the more common diagnoses, such as community-acquired pneumonia, does not improve the patient's condition. In this case, the patient was non-verbal. That is a barrier to knowing how the patient feels and obtaining a proper review of the system history. The patient did not present with the classic signs of proximal muscle weakness, and an accurate muscular exam was not possible, leading to further complexity in the diagnosis. The patient in our case had to be intubated and also had a cardiac arrest, for which an extensive workup is necessary, including a rheumatologic workup [12]. The positive test rest results of CPK, aldolase, and ANA directed us toward diagnosing polymyositis. It is essential for clinicians to know and realize the severity of some rheumatologic diseases; in this case, the treatment is straightforward with steroids and life-saving.

## Conclusions

This case report illustrates that clinicians must educate patients about the risk of developing respiratory failure, sometimes requiring intubation if they have polymyositis. The risk is higher for patients less than 45 years of age. Awareness of possible complications can lead to earlier diagnosis and prevent ER visits and complicated ICU stays. 
